# Notes and News

**Published:** 1904-10-22

**Authors:** 


### NOTES AND NEWS.

His Majesty the King has consented to give his patronage to the Sanitary Institute. The Institute was founded in 1876, and it is carrying on a large work in teaching and examining in hygiene and sanitary science, both in the United Kingdom and in other parts of the Empire. It maintains in London a permanent Museum of Sanitary Appliances, open free to the public. Its members and associates number over 3,000.

Lectures will be given under the auspices of the Childhood Society in the Library of the Sanitary Institute, 72 Margaret Street, W., on Thursday, October 27th, on "Physical Condition of Working Class Children," by T. J. Macnamara, LL.D., M.P. On Thursday, November 10th, on "Mental Hygiene in Childhood," by T. B. Hyslop, M.D., M.R.C.P., Edin. On Thursday, November 24th, on "Education of Girls," by Miss M. E. Findlay, B.A. A discussion on "Physical Deterioration" will take place on October 20th.

At the last meeting of the Norfolk and Norwich Hospital, a most interesting discussion took place on the question of the desirabilty of admitting ladies on the Board of Management. Lord Lindley established the legal eligibility of lady governors to occupy such a position in an able manner, and the Dean of Norwich most eloquently established their claims from the standpoint of capacity and suitability. If the words of Dr. Barton are remembered in years to come, when this question has passed, as it must, out of the region of dispute, they will certainly cause a smile. To-day his standpoint is certainly belated. He said that the Norfolk and Norwich Hospital had a great reputation for management to lose, and he described the proposal as "upsetting the present management," and as "revolutionary." Major Edwards went as far as to call the suggestion "an insult to the Board of Management." These were the strongest remarks in opposition to the resolution, which, on being put to the vote, was carried by 30 votes, with 20 against it. The arguments at this meeting in favour of lady governors sitting on the Board of Management were undoubtedly convincing, as on a previous occasion the same proposal was defeated by an overwhelming majority.

It is reported that beri-beri is very prevalent in the Japanese Army, and that the beseigers of Port Arthur are the greatest sufferers.

New York State traces a consistent autumnal increase of typhoid to contamination from the wells on farms where so many people pass their holidays. It would be a very simple matter, and no hardship, to insist upon the use of earth closets in country places, and the abolition of the dangerous privy.

The question of part payment by out-patients of the London Hospital is not slumbering amongst the medical men in the East End, who object to the arrangement. They have not succeeded in bringing the authorities at the hospital to their way of thinking, and so they contemplate an endeavour to bring definite notice from without upon the grievances they complain of. One proposal is to appeal to the giving public, and the other is to endeavour to enlist the help of the General Medical Council.

At a recent meeting of the Governors of the Windsor Royal Dispensary and Infirmary, it was decided to empower the Site Committee to negotiate for the purchase of a site on Crown lands opposite the Combermere Barracks. The position selected seems eminently suitable for Windsor's new hospital, and the arguments brought forth in favour of a large and airy site, adjacent to but not actually in the centre of the town, is a further witness to the fact that education in matters of hygiene is rapidly spreading. A few years ago it would have been improbable that so representative a gathering of persons of local standing, capable of discussing in so intelligent a manner the pros and cons of a hospital site, would have met together with one accord, as was the case at Windsor recently.

A satisfactory method of ventilating railway carriages has not yet been discovered in this country. Under the present systems there is either too much fresh air or too little. The passenger in command of the window is usually at variance with the rest of the occupants of the carriage as to the amount of air necessary for comfort; and in any case, those who sit with faces towards the engine, must meet a steady gale of wind from open windows; whilst those sitting on the opposite side of the carriage are frequently in need of more air. There is no doubt that ventilation, depending upon windows at the side of a fast-going vehicle, is most unsatisfactory. The Pennsylvania Railway have been making experiments with two hoods placed at diagonally opposite corners of the carriages through which air is introduced into down takes underneath the hoods, to spaces below the car-floor and (in winter over the heating apparatus) into the car. By this means an attempt has been made to pass 60,000 cubic feet of air per hour through the carriages. This method is said to be a vast improvement on open-window ventilation, but we have not yet heard the personal testimony of a passenger. Nevertheless, English travellers who have experienced the overheating of the London and North-Western carriages in winter, and the draughts in those of the South-Western Railway, cannot but wish these enterprising companies would study improved systems.

